# The Role of Bioactive Compounds from Dietary Spices in the Management of Metabolic Syndrome: An Overview

**DOI:** 10.3390/nu14010175

**Published:** 2021-12-30

**Authors:** Dana Hasan Alkhatib, Abdul Jaleel, Maryam Naveed Muhammad Tariq, Jack Feehan, Vasso Apostolopoulos, Leila Cheikh Ismail, Lily Stojanovska, Ayesha S. Al Dhaheri

**Affiliations:** 1 Department of Nutrition & Health Sciences, College of Medicine and Health Sciences, United Arab Emirates University, Al Ain 15551, United Arab Emirates; danakhatib@uaeu.ac.ae (D.H.A.); 201250430@uaeu.ac.ae (M.N.M.T.); lily.stojanovska@uaeu.ac.ae (L.S.); 2 Department of Integrative Agriculture, College of Agriculture and Veterinary Medicine, United Arab Emirates University, Al Ain 15551, United Arab Emirates; abdul.jaleel@uaeu.ac.ae; 3 Institute for Health and Sport, Victoria University, Melbourne, VIC 3001, Australia; jack.feehan@vu.edu.au (J.F.); vasso.apostolopoulos@vu.edu.au (V.A.); 4 Immunology Program, Australian Institute of Musculoskeletal Science (AIMSS), Melbourne, VIC 3021, Australia; 5 Department of Clinical Nutrition and Dietetics, College of Health Sciences, University of Sharjah, Sharjah 27272, United Arab Emirates; lcheikhismail@sharjah.ac.ae; 6 Nuffield Department of Women’s & Reproductive Health, University of Oxford, Oxford OX1 2JD, UK

**Keywords:** herbal therapy, bioactive compounds, natural products, metabolic syndrome, diet therapy

## Abstract

Metabolic syndrome (MetS) is a combination of physiologically dysregulated parameters that can include elevated fasting blood glucose, high blood pressure, central obesity, increased triglyceride levels, insulin resistance, diabetes, elevated low density lipoprotein levels, and reduced high density lipoprotein levels in the blood. Effective clinical management of MetS is critical as it is strongly associated with long lasting and fatal complications in patients. Alongside standard care of lifestyle changes and medication, dietary supplements derived from herbal resources could be an alternative therapeutic strategy that is safe, efficient, culturally acceptable, and has few side effects. Of the dietary supplements, spicy foods have always been considered a great source of functional bioactive compounds. Herbal therapy is broadly used in many countries as a treatment or as a preventive measure in the management of MetS risk factors, including blood glucose, blood pressure, and blood lipid levels. Herein, an attempt is made to evaluate the recent studies in the management of MetS with herbal alternatives, and to explore the possibility of their use as therapeutic treatments or supplements.

## 1. Introduction

### 1.1. Metabolic Syndrome

From the early 1920s to the late 1980s, researchers have noted the relationship between upper body obesity, diabetes, and hyperlipidemia, and an increased risk of cardiovascular disease (CVD) [1,2] with the term “metabolic syndrome” (MetS) coined to describe the cluster of abnormalities in 1977. It was first described by Haller et al. [3], when discussing the association between diabetes mellitus type-2 (type-2 diabetes), obesity, hyperlipoproteinemia, hyperuricemia, and hepatic steatosis, as cumulative risk factors for atherosclerosis. In the same year, it was also used to describe the correlation of obesity, gout, type-2 diabetes, and hypertension with hyperlipoproteinemia [4]. One year later, Phillips et al. [5] developed the concept further, suggesting that risk factors of myocardial infarction including glucose intolerance, hyperinsulinemia, hypercholesterolemia, hypertriglyceridemia, and high blood pressure are associated with heart disease, aging, and obesity. MetS, or ‘syndrome X’, was first introduced in 1988 as a cluster of related abnormalities, including hyperglycemia, dyslipidemia, and high blood pressure that could lead to CVD. It was suggested that all of these abnormalities were possibly caused by underlying insulin resistance [6]. Different names, such as MetS, American syndrome, syndrome X, and insulin resistance syndrome, have been used to describe the gathering of abnormalities that commonly lead to CVD and type-2 diabetes [7]. Recently, MetS in children has been redefined by Flemming et al. [8] with ways of early diagnosis of metabolic syndrome.

### 1.2. Metabolic Syndrome Diagnostic Criteria

The definitive importance of MetS is due to its ability to identify individuals who are at high risk of type-2 diabetes and CVD [7]. Therefore, numerous studies have attempted to outline a diagnostic criteria for MetS. In 1999, the World Health Organization (WHO) focused on insulin resistance, diabetes, or impaired glucose tolerance as the crucial components of MetS, with at least two of the following present: hypertriglyceridemia, high blood pressure, low high density lipoprotein (HDL) levels, obesity, and microalbuminuria [9]. In the same year, the European Group for the Study of Insulin Resistance (EGIR) altered the WHO criteria by eliminating people with type-2 diabetes; however, the presence of hyperinsulinemia was still required. Even though waist circumference is used for measuring obesity; however, different cutoff values were considered for the other variables such as central obesity, hypertension, dyslipidemia, and impaired fasting glucose [10]. In 2001, the US National Cholesterol Education Program (NCEP): Adult Treatment Panel III (NCEP ATP III) excluded obligatory insulin resistance, instead considering hyperglycemia to be a diagnostic criterion along with the elevated triglycerides (TGs), low HDL, central obesity, and elevated blood pressure. On the other hand, the NCEP concluded that for an individual to be diagnosed with MetS, any three of the five diagnostic criteria listed by the NCEP ATP III must be present [11]. The International Diabetes Federation (IDF) amended the WHO definition by requiring the inclusion of central obesity criteria in the definition along with increased TG, high blood pressure, high fasting blood glucose (FBG), and reduced HDL [12].

A number of other parties have also refined the previous MetS diagnostic criteria: the American Heart Association/National Heart, Lung, and Blood Institute (AHA/NHLBI) proposed lowering the FBG cutoff point to ≥100 mg/dL as opposed to the ATP III FBG cutoff point (≥110 mg/dL). They also did not incorporate ethno-specific values for central obesity and did not consider central obesity as diagnostic criterion [13]. These differences in the MetS diagnosis criteria led to confusion amongst the research community and a meeting of the IDF Task Force on Epidemiology and Prevention; the National Heart, Lung, and Blood Institute (NHLBI); the American Heart Association (AHA); the World Heart Federation (WHF); and the International Atherosclerosis Society (IAS) to standardize the criteria. The consensus approved that there should not be a mandatory diagnostic criterion and that waist circumference (central obesity) should be the main screening tool. The meeting outcomes also concluded that the presence of three out of five risk factors would diagnose MetS, with the five criteria being elevated blood pressure, central obesity, elevated TG, elevated FBG, and reduced HDL [14]. MetS and its relation to dietary patterns were recently studied, and it was concluded that nutrition interventions need to be tailored to address the MetS occurrence in various ethnic groups [15].

### 1.3. Metabolic Syndrome Risk Factors

Diet, physical inactivity, age, sex, genetics, stress, and ethnicity are factors that could contribute to the development of MetS-associated risk factors. Many researchers have linked obesity and high waist circumference to CVD [16,17,18]. According to the WHO, obesity is a medical condition characterized by high accumulation of adipose tissue, which can lead to serious health issues [19]. This increase in fat tissue leads to a range of physiological changes, including insulin resistance and chronic inflammation [20]. The increase in the amount of adipose tissue increases tumor necrosis factor (TNF)α levels, which have secondary effects on a range of metabolic pathways and increases the level of cytokines in the blood. TNFα, through the action on its receptor, has been suggested to directly increase insulin resistance [21]. Another outcome of increasing adiposity is an increase in cytokines that regulate the balance between humoral and cellular immune responses. This could lead to imbalances in the immune response and inflammation, which could lead to atherosclerosis [22].

Increased blood pressure is considered to be one of the key underlying causes of CVD [23], as well as a risk factor that could cause strokes, dementia, CVD, and chronic kidney disease [24,25,26]. Decreasing blood pressure by 5 mmHg has been shown to decrease the likelihood of ischemic heart disease (IHD) by 21% and stroke by 34%, while also reducing the probability of heart failure, dementia, and mortality from CVD [24].

Dyslipidemia is another important cause of CVD [27]. A meta-analysis showed that elevated TG levels in the blood could lead to CVD and other health complications [28]. In addition, low HDL and high low density lipoprotein (LDL) levels are correlated with an increased risk of CVD [29,30]. Borg et al. (2011) showed a strong relationship between glycated hemoglobin (HbA1c) and CVD, where there was a correlation between high blood glucose and an increased risk of CVD in patients with diabetes [31]. In a study of 237,468 participants, it was shown that prolonged abnormal high levels of glucose in the blood led to 1661 strokes and 816 IHD events [32]. Hence, MetS and CVD are strongly related and must be considered as a central part of management. A number of studies have called the effects of MetS risk factors into question and evidenced the direct effects of these risk factors on CVD, quality of life, and morbidity rate [33,34,35].

Prolonged periods of stress may also play a negative role in the development of MetS by disturbing endocrine homeostasis [36]. Stress can disturb the hypothalamic–pituitary–adrenal (HPA) axis [37], leading to an increase in cortisol levels, further exacerbating the alterations associated with MetS including increased blood pressure [38], blood glucose levels [39], insulin resistance [40], dyslipidemia [38,41], and visceral adiposity [42]. As established, these factors all contribute to the risk of CVD, type-2 diabetes, and stroke [34,43].

## 2. Search Methodology

For the collection of relevant literature, an extensive article search was carried out through various electronic databases, namely Google Scholar databases, MEDLINE (PubMed), Scopus, Web of Science, ReseachGate, and books through available UAEU Library resources. A comprehensive search strategy was carried out by giving relevant keywords and their combinations. Search terms used were ‘Metabolic Syndrome management’ OR ‘Herbal therapies’ OR ‘dietary spices’ AND ‘Metabolic Syndrome’ OR ‘Diagnosis of Metabolic Syndrome’ OR ‘Ginger’ AND ‘Herbal therapies’ OR ‘edible spices’ OR ‘Herbal Therapeutics’ OR ‘Cinnamon’ OR ‘Black Seed’ AND ‘MetS management’ OR ‘Fenugreek’ OR ‘Saffron’ OR ‘Cardamom’ OR ‘Turmeric. The resulted research articles, review articles, books, and theses relevant to our studies until 2021 were explored and studied thoroughly, and important literature was included in the present study.

## 3. Herbal Approaches to Management in Metabolic Syndrome

Management of MetS requires individualized treatment, centered on lifestyle changes in the first instance [44,45,46]. As MetS is characterized as a group of factors elevating the risk for CVD and type-2 diabetes, management should begin by addressing each factor individually. This can be achieved by a variety of means, including increasing physical activity levels, improving diet, and pharmacological management. Classically, this includes the use of medications; physical activity; and diet to improve blood pressure, HDL, TG, and glucose levels [44,47].

While the western means of managing these key risk indicators such as medication and exercise prescription are well established, in eastern cultures, the use of herbs and spices is common and may provide important advantages that could be used alongside medical approaches to manage MetS [48,49]. Herbal or alternative therapies are prescribed to relieve and treat symptoms of different diseases [49]. In fact, in 41 animal studies, herbal therapies led to weight loss or significant restriction of weight gain [50]. Moreover, individuals with a higher income and education were more likely to be taking herbal products with the goal of overall health improvement [51], and other studies have reported significant use of herbal therapies among a range of ethnic groups [52,53]. A number of herbs and spices have been shown to be effective in managing key risk factors of MetS, including obesity/abdominal obesity, lipid profiles, hypertension, FBG, and insulin sensitivity [48,50,54]. Furthermore, an efficient decrease in the waist-to-hip ratio and waist circumference were reported in studies that used one or more herbal/spice extracts, including the use of *Zingiber officinale* [55,56], and others have reported decreased appetite in obese individuals after regular consumption of some herbs leading to weight loss [57,58]. Illustrating the clinical use of some herbs and spices, Table 1 contains 13 studies conducted in humans, and the effects of herbs and spices on MetS risk factors are noted.

### 3.1. Common Herbal Therapeutics, and Their Effect on Metabolic Syndrome Indicators

**Figure 1:**
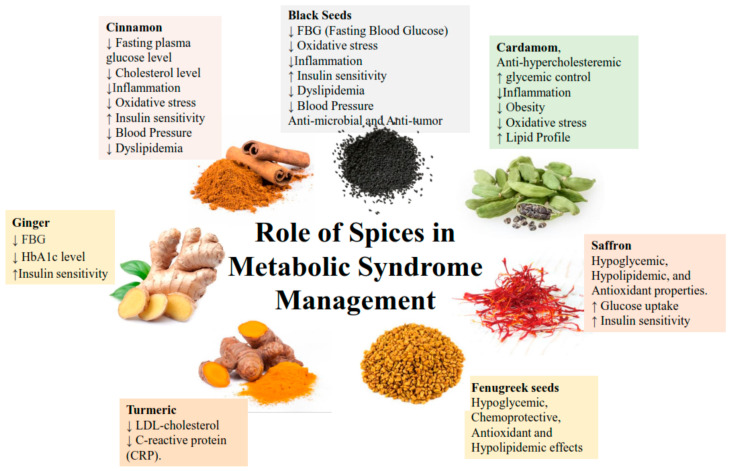
Common spices used in food, and their impact on MetS risk factors.

Ginger (*Zingiber officinale*), cinnamon (*Cinnamomum*), and black seed (*Nigella sativa*) are annual plants that have been widely used internationally, particularly in India, Europe, and Arabian countries [69]. These spices are widely used for food preparation and medicinal purposes, as a treatment for many conditions including diabetes, asthma, hypertension, inflammation, cough, bronchitis, headache, eczema, fever, dizziness, and influenza. Additionally, black seed is used as a diuretic, lactagogue, and vermifuge, whilst ginger and cinnamon are used as anti-tumor agents, with cinnamon also used to decrease muscle soreness in athletes [69,70,71,72,73,74,75]. These spices are also used in food as aromatic spices, carminatives, and condiments (Figure 1).

Chemical analysis reveals that specific herbs and spices have different chemical compositions and as such have different active components like essential oils with medicinal values [76]. Importantly, different soil types and geographical locations lead to significant differences in nutrient composition [77].

#### 3.1.1. Ginger

Ginger has a moisture content of 15%, with 5% protein, 3.72% fat, 38.35% carbohydrates, 25.5% soluble fiber, 23.5% insoluble fiber, and 3.85% total ash [78]. In addition, ginger contains many different vitamins and minerals such as vitamin C, calcium, phosphorous, zinc, and iron [79,80], and is a good source of antioxidants due to its polyphenol content, particularly tannins and flavonoids [78]. Neither the active ingredient nor mechanism of action of ginger is known. It is suggested that some of the active ingredients of ginger are 6-paradol and 6-shogaol, these chemicals give ginger its pungent smell and taste and are believed to impart its anti-glycemic effect. A study noted that 6-paradol and 6-shogaol stimulate glucose usage by adipocytes and myotubes in high-fat diet-fed mice. This effect was accredited to an elevation in adenosine monophosphate-activated protein kinase (AMPK) phosphorylation [81]. Another study showed that the activity of (S)-(8)-gingerol was correlated with an elevation of the surface expression of the glucose transporter type 4 (GLUT4) protein, which is responsible for glucose uptake in muscle and adipose tissues [82].

The effect of ginger on glycemic parameters has variable outcomes. A meta-analysis of studies concluded that ginger consumption in different forms (tablet, capsules, powder, or rhizomes) substantially lowered FBG and TG, while it also elevated HDL levels [83]. Further, in a randomized double-blind placebo-controlled clinical study, the effects of a daily intake of 2 g of ginger powder in 40 obese women was assessed. It was shown that ginger treatment had a non-significant reducing effect on serum glucose, a slight positive effect of ginger powder consumption on serum glucose, and a significant effect on TG when compared to placebo [84]. Another randomized double-blind placebo-controlled trial conducted in individuals with type-2 diabetes for 2 months examined the effects of ginger powder on glycemic parameters and found that ginger significantly increased insulin sensitivity, but had no effect on FBG and HbA1c [85]. So, while ginger appears to have some impact on glycemic control, it is unclear what the specific outcomes are in humans.

There is less convincing evidence for ginger as an antihypertensive agent. A randomized controlled trial randomly distributed 204 individuals living with type-2 diabetes to four intervention groups, who received 1 g of saffron powder or 3 g of cinnamon, cardamom, or ginger powder. The participants were asked to consume the spices with three glasses of black tea every day. The control group consumed only three glasses of tea without any spice powder for 8 weeks. It was clear that none of the spice powders had a significant effect on improving blood pressure [69]. A review came to the same conclusion regarding the antihypertensive effect of ginger, concluding it had no significant effect on blood pressure. However, the same review also reported that ginger could be offered as a natural alternative dietary supplementation to anti-hypertensive factors in animal studies. However, there is not inadequate evidence to support the same outcome in human studies [86].

While ginger may offer little benefit to improving blood pressure, it may have a stronger effect on blood lipid profile [87]. A meta-analysis study showed that ginger consumption (tablet, capsules, powder, or rhizomes) had a significant effect on decreasing TG and elevating HDL [83]. In a randomized double-blind placebo-controlled trial, it was noted that ginger significantly decreased serum LDL and TGs in participants with type-2 diabetes [85]. Whilst another showed that a 2 g ginger treatment significantly reduced TG levels in the blood compared to the placebo group in obese women [84]. Moreover, a randomized double blinded study examined the effects of 3 g of ginger powder for 45 days on CVD patients in Iran, concluding that ginger powder caused a significant reduction in TGs, cholesterol, LDL, and very low-density lipoprotein (VLDL) levels in the blood [88].

Ginger has also been suggested to have a beneficial effect on weight [89]. A pilot study examining the short-term effects of hot ginger beverages on feelings of satiety, energy expenditure, and metabolic risk factors in overweight men found no significant effect on blood glucose, insulin, lipid profile, or inflammatory markers, but did improve thermogenesis and increased feeling of satiety, suggesting a potential role in managing weight [90]. However, a larger trial in 2014 examining the effects of each cinnamon, cardamom, saffron, and ginger powder for 8 weeks on diabetic individuals concluded that none of the spices powders had a significant effect on improving weight or waist circumference [69]. Another study examined the influence of ginger combined with high intensity training on inflammation. Thirty overweight females consumed 3 g/day of ginger in the first group; in the second group, participants consumed 3 g/d of ginger + high intensity training; and in the third group, participants consumed 3 g/d of placebo + high intensity training for 10 weeks. This study showed that interval exercise, by itself or combined with a ginger supplement, improved the maximum oxygen consumption but did not significantly lower the body fat percentage or the waist-to-hip ratio in the participants [91].

#### 3.1.2. Cinnamon

Cinnamon (*Cinnamomum*) contains 3.5% crude protein; 4% crude fat; 52% carbohydrates; 33% crude fiber; and 2.4% total ash; as well as several different vitamins and minerals including potassium, copper, phosphate, zinc, and iron [92]. The key active ingredients of cinnamon are cinnamaldehyde and cinnamic acid [93]. Cinnamaldehyde and some of its derivatives, 2′-hydroxycinnamaldehyde and 2′-benzoyl-oxycinnamaldehyde, can increase the level of reactive oxygen species (ROS) and in induce apoptosis through inhibition of proteasome activity in carcinogenic cells, making them more prone to oxidative stress [94,95]. In CVD, cinnamaldehyde and cinnamic acid together have the ability to produce nitric oxide and both have anti-inflammatory effects, of which are known to slow atherogenesis [96]. The antioxidant properties of cinnamon result from the activity of the aromatic oil eugenol, which stops peroxynitrite-induced nitration, and cinnamon also includes a number of active polyphenols that are also considered to improve insulin sensitivity [97] and reduce inflammation [98].

There is evidence to suggest that cinnamon has the capacity to improve glucose control in humans [99]. Indeed, a study examined the effects of cinnamon in people living with diabetes at three different doses: 1, 3, and 6 g/day for 40 days, finding it decreased FBG levels significantly [72]. Likewise, a meta-analysis of 10 randomized controlled trials of 543 diabetic patients also found that consuming cinnamon at a dose from 120 mg/day to 6 g/day for 4 months significantly reduced FBG levels [100]. However, this was not the conclusion of another meta-analysis, which found no significant effect on HbA1c or FBG [101].

There is also support for cinnamon as an antihypertensive agent. In a randomized, placebo-controlled, double-blind clinical trial, 58 people with type-2 diabetes were randomly assigned to consume either 2 g/day of cinnamon or placebo for 12 weeks. The study showed a significant decrease in both systolic and diastolic blood pressure [102]. This finding was supported by a systematic review published on the effects of cinnamon on blood pressure in individuals with diabetes mellitus, reporting that short-term consumption of cinnamon is linked to a significant decrease in systolic and diastolic blood pressure [103].

The evidence on cinnamon and lipid profile is less clear. A study evaluating the effect of cinnamon in individuals with diabetes for 40 days showed that cinnamon powder significantly decreased TG, LDL, and total cholesterol levels in the blood [72]. This was supported by a meta-analysis that found that cinnamon significantly lowered total cholesterol, LDL, and TG, and improved HDL concentrations but had no significant effect on HbA1c [100]. However, another meta-analysis of randomized controlled trials of cinnamon was published and concluded that cinnamon consumption did not improve any lipid parameters significantly [101].

Another study tested the effects of a cinnamon, chromium, and magnesium-formulated honey on blood glucose, weight, and lipid profile in diabetic individuals. Following the 40-day intervention, there was no change to FBG or HbA1c, and no significant improvement of lipid profile or weight; however, the authors did report a tendency towards increased HDL and decreased systolic blood pressure in the treatment group [104]. Another RCT in which 30 participants supplemented breakfast cereal with cinnamon found that cinnamon moderated postprandial glucose response in normal weight and obese adults, potentially assisting with weight management [105]. Another study evaluating the effect of cinnamon on insulin sensitivity in diabetic adults found significant improvements in the FBG level, lipid profile, blood pressure, and body fat percentage, as well as increased lean body mass for participants who consumed cinnamon [97].

#### 3.1.3. Black Seed

Black seed contains 26.7% protein, 28.5% fat, 24.9% carbohydrate, 8.4% crude fiber, and 4.8% total ash. Black seed also contains copper, phosphate, zinc, iron, and essential (volatile) and fixed oils. Black seed essential oil contains a major bioactive component, thymoquinone (TQ; 30–48%) [70]. TQ is the principle and active ingredient of black seed. It is a chemical compound that is known for its therapeutic potential, and the anti-inflammatory, hypoglycemic, and antioxidant properties of black seed reported in the literature have been attributed to TQ [106,107]. Black seed can be added to different types of food such as tea, coffee, casseroles, or breads, and is also used in canning. Additionally, its extract is used in wine [108].

Black seed has been shown to offer beneficial effects on cardiometabolic parameters in adults. A study found that supplementation with 3 g/day of black seed oil significantly improved FBG, HbA1c, total cholesterol, TG, HDL, and LDL levels in the treatment group when compared to the placebo group after 12 weeks of consumption [109]. Another study showed that taking 2 g/day of black seeds for 3 months decreased FBG, 2-h post-prandial glucose (2-hPG), HbA1c, and increased insulin sensitivity without any renal or hepatic side effects in individuals with type 2 diabetes mellitus [110]. Additionally, it has been reported that black seed has anti-diabetic and hypoglycemic activity as the components of black seed decrease oxidative stress and thus preserve pancreatic beta cell integrity [111].

Black seed has also been evaluated in individuals with hypertension [112]. A double-blind randomized controlled trial enrolled 62 patients with MetS and found that supplementation with 3 g/day of black seed powder for 3 months significantly reduced systolic blood pressure [113]. This was echoed in another study of 159 participants evaluating a 500 mg/day black seed powder supplement over 6 weeks. The participants also received oral hypoglycemic and antihypertensive drugs and a lifestyle intervention including a low-fat diet and physical activity such as walking (60–90 min per day). This black seed intervention showed a significant decrease in blood pressure when compared to the placebo group [114]. Another study in patients with mild hypertension evaluated two doses of black seed: 100 mg and 200 mg of black seed extract twice per day, over 8-weeks, finding that it decreased systolic and diastolic blood pressure in a dose-dependent manner [115].

Black seed may also offer benefits to lipid profiles. In fact, 1 g/day intervention of black seed powder for 12 weeks increases HDL levels, and 2 g/day decreases total cholesterol, LDL concentrations, and TG levels. Increasing the dosage from 2 to 3 g/day did not further improve the lipid profiles, although the most effective dose was between 2–3 g/day to improve total cholesterol, TG, LDL, and HDL concentrations [116]. A randomized controlled trial investigating the effect of black seed consumption on menopausal women for 2 months showed decreases in total cholesterol, LDL, and TG, and an elevation in HDL [106]. Similarly, 2 g/day of black seed for 4 weeks significantly lowered total cholesterol, LDL, and TG levels [117].

Unlike some of the other herbal therapies, black seed may also have the capacity to reduce obesity. One study evaluated a 3-month black seed intervention, finding a decrease in waist circumference in the treatment group [113]. Additionally, a double-blind randomized controlled found that consuming 1.5 g/day of black seed powder for 4 weeks significantly reduced waist circumference [108]. Another showed that 250 mg of black seeds for 6 weeks, along with dietary modification (cholesterol-free diet) and a walking program, provided protection against MetS and significantly lowered waist circumference [114]. A prospective randomized controlled study of 60 participants with MetS showed that 2.5 mL of black seed oil twice daily along with 10 mg of atorvastatin for 6 weeks decreased both weight and waist circumference compared to controls [118]. Another interventional prospective randomized controlled study of 161 participants taking 2.5 mL of black seed twice a day for 6 weeks showed that there was an improvement in body weight and BMI in both the treatment group and the placebo group, but the improvement was greater in the black seed group when compared to the placebo group [119]. A review on black seed suggested that it does not have a direct effect on reducing body weight, but instead effects food intake via anorexic effects [120]. There are, however, some contradictory findings. A randomized, double-blind, placebo-controlled study on diabetic patients found that ingestion of 3 g/day of black seed oil for 12 weeks does not cause a significant change in body weight [109]. Additionally, another study found an equivalent decrease in the body weight in both intervention and placebo groups [118].

### 3.2. Other Herbal Interventions

While the greatest body of research in key MetS parameters centers on ginger, cinnamon, and black seed, there are other candidates that have also been evaluated such as artichoke, allium sativum, and the many herbs with soluble fibres. However, we describe some other commonly used herbs including fenugreek, saffron, cardamon, and turmeric.

#### 3.2.1. Fenugreek

The culinary spice fenugreek (*Trigonella foenum graecum*) is a legume seed belonging to the Fabaceae family, which has been widely cultivated and produced in Mediterranean countries and Asia and is commonly used as alternative medicine in India and Iran. The chemical composition of fenugreek is primarily carbohydrate, being 47.4%, followed by protein 28.4%, crude fiber 9.3%, crude fat 7.1%, moisture 6.87%, and ash 3.28% [121]. Fenugreek is known to have hypoglycemic, chemoprotective, antioxidant, and hypolipidemic pharmacological effects [122,123,124,125]. Fenugreek contains alkaloids, flavonoids, salicylate, and nicotinic acid as active compounds, which are thought to impart its physiological effects [126,127,128]. In high fat-fed animals, fenugreek supplementations improved cholesterol levels, triglycerides, reduced fatty acid binding protein 4, and increased subcutaneous adipose tissue expression of adiponectin, but did not improve glucose tolerance [129]. Likewise, in high-fat diet-induced obese rats, fenugreek inhibited fat accumulation and reduced dislipidemia [130]. In healthy human subjects, fenugreek seeds and leaves reduced blood glucose levels considerably following high glucose dose or meal, and in diabetic patients, plasma glucose levels and cholesterol levels were significantly improved [131].

#### 3.2.2. Saffron

The costly spice saffron (*Crocus sativus*) belongs to the Iridaceous family and is a caroteinoid-rich spice that has been evaluated for its wide ranging medicinal actions including hypoglycemic, hypolipidemic, and antioxidant properties. It has been shown that saffron increases glucose uptake and improves insulin sensitivity [132], as well as inhibits platelet aggregation and membrane lipid peroxidation [133,134]. In a randomized, double blind, placebo-controlled study, supplementation of saffron tablets in 40 type-2 diabetic patients vs. 40 control subjects for 12 weeks, reduced waist circumference and malondialdehyde levels, although no changes were noted in blood glucose levels, lipid profiles, C-reactive protein, and pro-inflammatory marker tumor necrosis factor [135]. However, in a systematic and meta-analysis study of 15 randomized controlled trials involving 1139 subjects, saffron was shown to decrease glycemic indices including FBS, HbA1c, and fasting insulin, and increased quantitative insulin sensitivity check index [136].

#### 3.2.3. Cardamon

Cardamom, part of the Zingiberaceae family, comes as black (*Amomum subulatum* Roxburgh) or green (*Elettaria cardamomum* Maton), and is commonly used in culinary and traditional medicine practices in India and South Asia [69,137,138]. Cardamom has a high fiber content of approximately 22%, moisture 8%, protein 6%, total ash 4%, and oils (oleoresin and essential oil) [139]. In a rat model of high-fat-fed diet, cardamom was noted to have an anti-hypercholesteremic effect [138]. In addition, rats fed for 16 weeks with either corn-starch or high-carbohydrate high-fat diet with simple sugars and trans fats, black cardamon supplementation was more effective than green cardamon in regards to reversal of diet-induced changes, triglyceride levels, blood pressure, total body fat mass, and visceral adiposity [140]. In one human study, cardamom improved the lipid profile, but not glucose and insulin regulation [69], however, in another study, green cardamon decreased blood pressure, glycemic indices, and serum lipid levels [141].

#### 3.2.4. Turmeric

Turmeric (*Curcuma longa* L.) is obtained from the rhizome, part of the Zingiberaceae family, and it is known for its golden color [142]. It is native to South Asia and has been used as a herbal remedy due to its bioactive ingredients. Turmeric has three major curcuminoids or active ingredients, which include curcumin, demethoxycurcumin, and bisdemethoxycurcumin [59]. Curcumin or diferuloylmethane is the main bioactive ingredient, which makes up 60–70% of crude turmeric extracts and gives turmeric the yellow pigment with anti-inflammatory, antidiabetic, antioxidant, antibacterial, antiviral, and antifungal properties [60,61,142]. Turmeric contains approximately 12% moisture, 11% fat, 9% crude protein, 8% ash, and 3% crude fibre [62].

A randomized, double-blinded, placebo-controlled trial in 240 patients with prediabetes received either curcumin or placebo for 9 months. The findings revealed that curcumin supplementation significantly lowered the number of prediabetic individuals who eventually developed T2DM [63]. Studies on the effect of turmeric supplementation in patients with MetS found contradicting results on metabolic factors [64,65,66]. A recent systematic review and meta-analysis of randomized controlled trials on the metabolic benefits of curcumin supplementation in patients with MetS concluded that curcumin supplementation may improve some components of MetS such as fasting blood glucose, triglycerides, high-density lipoprotein cholesterol, and diastolic blood pressure, but not waist circumference and systolic blood pressure [142].

## 4. Conclusions and Future Prospective

The effects of ginger, cinnamon, and black seed (as well as fenugreek, cardamon, saffron, and turmeric) on key parameters related to MetS and CVD show promise as an adjunct nutritional intervention. They may also play key roles in the prevention of disease in high-risk individuals. However, before they can be effectively applied, additional studies are required to confirm their effect in humans, particularly evaluating those with MetS and CVD risk factors. Additionally, there is little information on the appropriate dosage or treatment regimens for these herbal interventions, and it is unclear what the mechanisms of their effect are in many instances. However, given their long-term use in traditional medical practice, with no evidence of adverse effects, they are likely already a safe adjunct to medical treatment, with likely benefits.

## Figures and Tables

**Table 1:** Studies and clinical trials on the effects of herbs and spices on metabolic syndrome risk factors.

Herbs	Target/Groups	Dose/Duration	Main Outcome	Study Reference
Slimax: extract of several plants: Hordeum vulgare, Polygonatum multiflorum, Dimocarpus longan, Ligusticum sinense, Lilium brownie, and Zingiber officinale	Healthy participants	6 weeks	Significant decrease in body weight and body mass index (BMI).	[55]
			Significant reduction in waist and hip circumference.	
Herbal supplement: (Ma Huang & Guarana)	Overweight participants	72 mg of ephedra and 240 mg of caffeine for 8 weeks.	Significant decrease in body weight and total body fat.	[56]
	-Control group (*n* = 24)		Significant reduction in hip and waist circumference.	
	-Intervention group (*n* = 24)			
A compound of *Aralia mandshurica* (A) and *Engelhardtia chrysolepis* (E) extracts called ARALOX	Obese non-diabetic women, *n* = 32	450 mg of *Aralia mandshurica* (A) and 450 mg of *Engelhardtia chrysolepis* (E) per day for 15 weeks.	Decrease in total body weight and fat weight.	[59]
	-Control group: Diet + placebo, *n* = 16		Reduction in plasma TGs.	
	-Intervention group:			
	Diet + compound, *n* = 16			
White bean extract *(Phaseolus vulgaris)*	Obese adults	3000 mg per day of each for 8 weeks.	Weight reduction in the intervention group.	[60]
	-Control group: placebo, *n* = 25		Decrease in plasma TGs.	
	-Intervention group: white bean extract, *n* = 25			
Turmeric (*Curcuma longa* L.)	Prediabetic adults	750 mg per day of each for 9 months.	16.4% of subjects in the placebo group were diagnosed with type 2 diabetes mellitus.	[61]
	-Control group: placebo, *n* = 25		None of the participants from the *Curcuma longa*-treated group were diagnosed with type 2 diabetes mellitus.	
	-Intervention group: *Curcuma longa*, *n* = 25			
Korean red ginseng (KRG) (*Panax ginseng*)	Overweight participants	6 g per day of each for 12 weeks.	No change in HbA1c in both groups.	[62]
	*n* = 19, with well-controlled type 2 diabetes		Intervention group maintained good glycemic control and improved plasma glucose and plasma insulin regulation.	
	-Control group: placebo, *n* = 9			
	-Intervention group: KRG, *n* = 10			
Bitter lemon (*Momordica charantia*)	Newly diagnosed with diabetes adults,	3 g per day of each for 12 weeks.	There was no significant effect on mean FBG, total cholesterol, and weight in both groups.	[63]
	-Control group: placebo, *n* = 20			
	-Intervention group: *Momordica charantia*, *n* = 20			
Cinnamon (*Cinnamomum*)	Participants diagnosed diabetes mellitus type 2	3 g per day of each for 16 weeks.	The cinnamon extract has a moderate effect in reducing fasting plasma glucose concentrations in diabetic patients.	[64]
	-Control group: placebo, *n* = 39			
	-Intervention group: cinnamon powder, *n* = 40			
A combination of *Cissus quadrangularis* (CQ) and *Irvingia gabonensis* (IG)	Overweight and obese participants	Intervention group: 300 mg CQ + 500 mg IG = 800 mg of compound per day.	Significant reduction in Cholesterol and LDL of FBG levels.	[65]
	-Control group: placebo, *n* = 36	Control group: 800 of placebo per day.	Significant decrease in body weight, body fat percent, and waist size in both groups.	
	-Intervention group: compound of CQ and IG, *n* = 36	Duration: 10 weeks		
*Terminalia arjuna* tree-bark powder	coronary heart disease (CHD) patients	Group I: placebo capsules;	Significant antioxidant action in the vitamin E group and *T. arjuna* tree group.	[66]
	Group I: control group, *n* = 35	Group II: vitamin E capsules 400 units per day;	Significant hypo-cholesterolemic effect in the *T. arjuna* tree group.	
	Group II: vitamin E group, *n* = 35	Group III received finely pulverized *T. arjuna* tree bark-powder (500 mg) per day		
	Group III: *T. arjuna* tree bark-powder group, *n* = 35	for 30 days.		
Ginger (*Zingiber officinale*)	Diabetic adults	3 g of each per day	Reduction in FBS and HbA1c.	[67]
	-Control group: placebo, *n* = 44	for 8 weeks.	Improvement in insulin resistance.	
	-Intervention group: ginger powder, *n* = 44			
Black seed (*Nigella stevia*) and turmeric (*Curcuma longa* L.)	Males with MetS *n* = 250 (randomly distributed	Black seeds (1.5 g/day)	Black seeds reduced lipids and FBG, while turmeric reduced LDL-cholesterol and C-reactive protein (CRP).	[68]
	-Control group:	Turmeric (2.4 g/day)		
	Placebo group, *n* = 64	combination (900 mg Black seeds and 1.5 g Turmeric/day)		
	-Treatment group:	placebo (2 g)		
	-Turmeric group, *n* = 62	for 8 weeks.		
	-Black seed group, *n* = 62			
	-Combination group, *n* = 62			
Cinnamon, cardamom, saffron (*Crocus sativus*) and ginger (*Zingiber officinale*)	Type 2 diabetes participants	For 8 weeks,	Significant beneficial effects on cholesterol, but not on measures of glycemic control, oxidative stress, and inflammation.	[69]
	-Control group:	three glasses of black tea and either 3 g/day of cardamom, or cinnamon, or ginger, or 1 g saffron. Control group received three tea glasses without any treatment.		
	Placebo, *n* = 39			
	-Treatment groups:			
	Cinnamon, *n* = 40			
	Cardamom, *n* = 42			
	Saffron, *n* = 42			
	Ginger, *n* = 41			

## Data Availability

Data sharing not applicable.
